# Supplementary material for: The Sugar Metabolic Model of Aspergillus niger Can Only Be Reliably Transferred to Fungi of Its Phylum
Source: J Fungi (Basel). 2022 Dec 17;8(12):1315. doi: 10.3390/jof8121315 (PMC9781776; doi:10.3390/jof8121315)
Supplement: Supplementary file 1 [file jof-08-01315-s001.zip › jof-2056969-supplementary/Supplementary Figure S1.pdf]

Supplementary Figure S1. Expression profiles of sugar metabolism-related genes in six fungi during their growth on diverse monosaccharides. Fig. **A** showing microarray expression profiling of genes involved in sugar meatabolic pathways of *A. niger*. Fig. **B-F** showing expression profiling (based on RNA-seq data) of genes involved in sugar meatabolic pathways of *A. nidulans, P. subrubescens, T. reesei, P. chrysosporium,* and *D. squalens,* respectively.
The blue color from light to dark indicates a gene expression level from low to high. Selected genes with specific sugar induced expression patterns are highlighted in blue on the heatmap, and their gene IDs and the associated pathways are displayed. Genes highlighted in red in Fig. **A** indicate they lack the corresponding expression value in previous microarray data, but these genes were predicted in the latest *A. niger* NRRL3 genome.

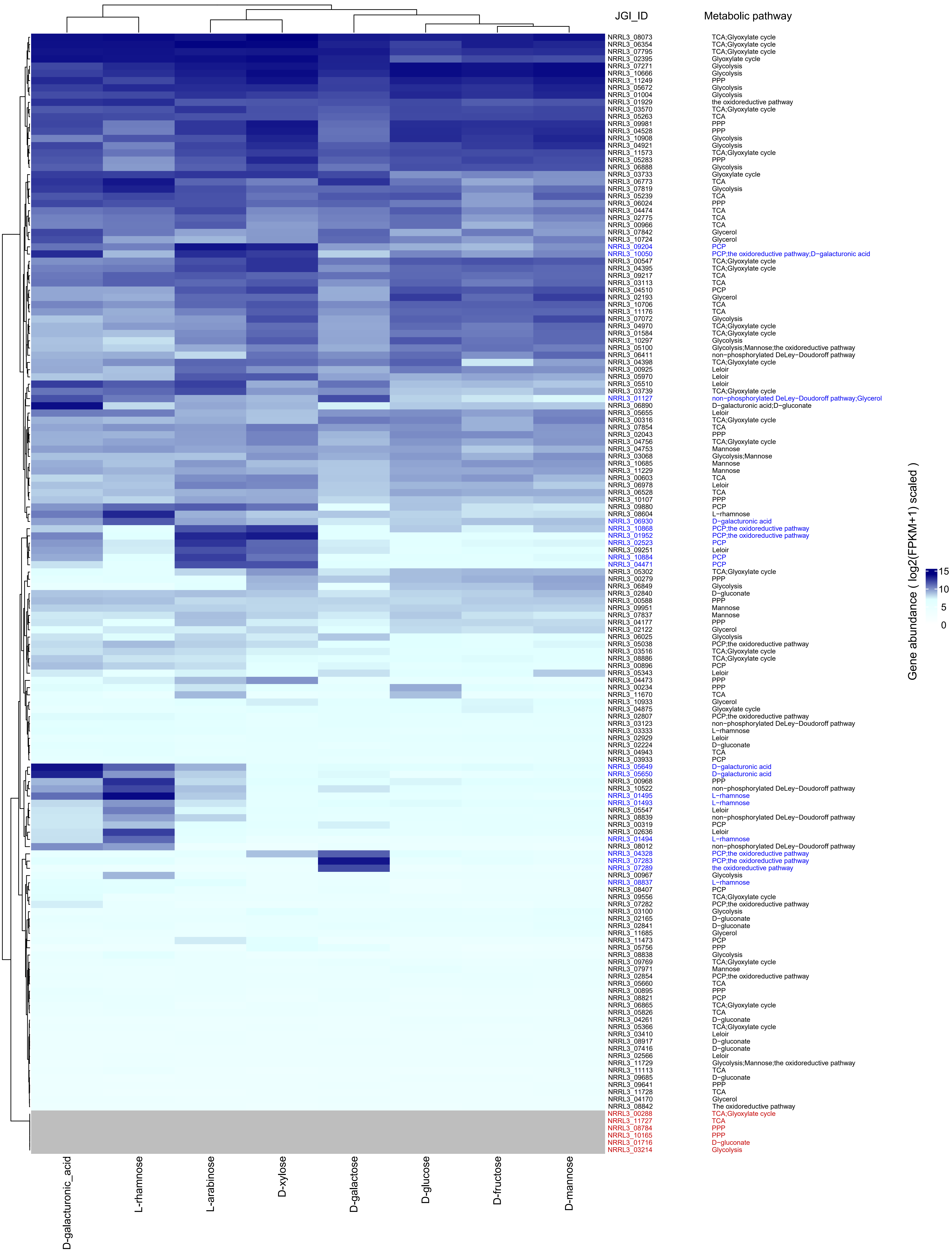

Supplementary Figure S1. A: Microarray expression profiling of genes involved in sugar metabolic pathways of *A. niger*.

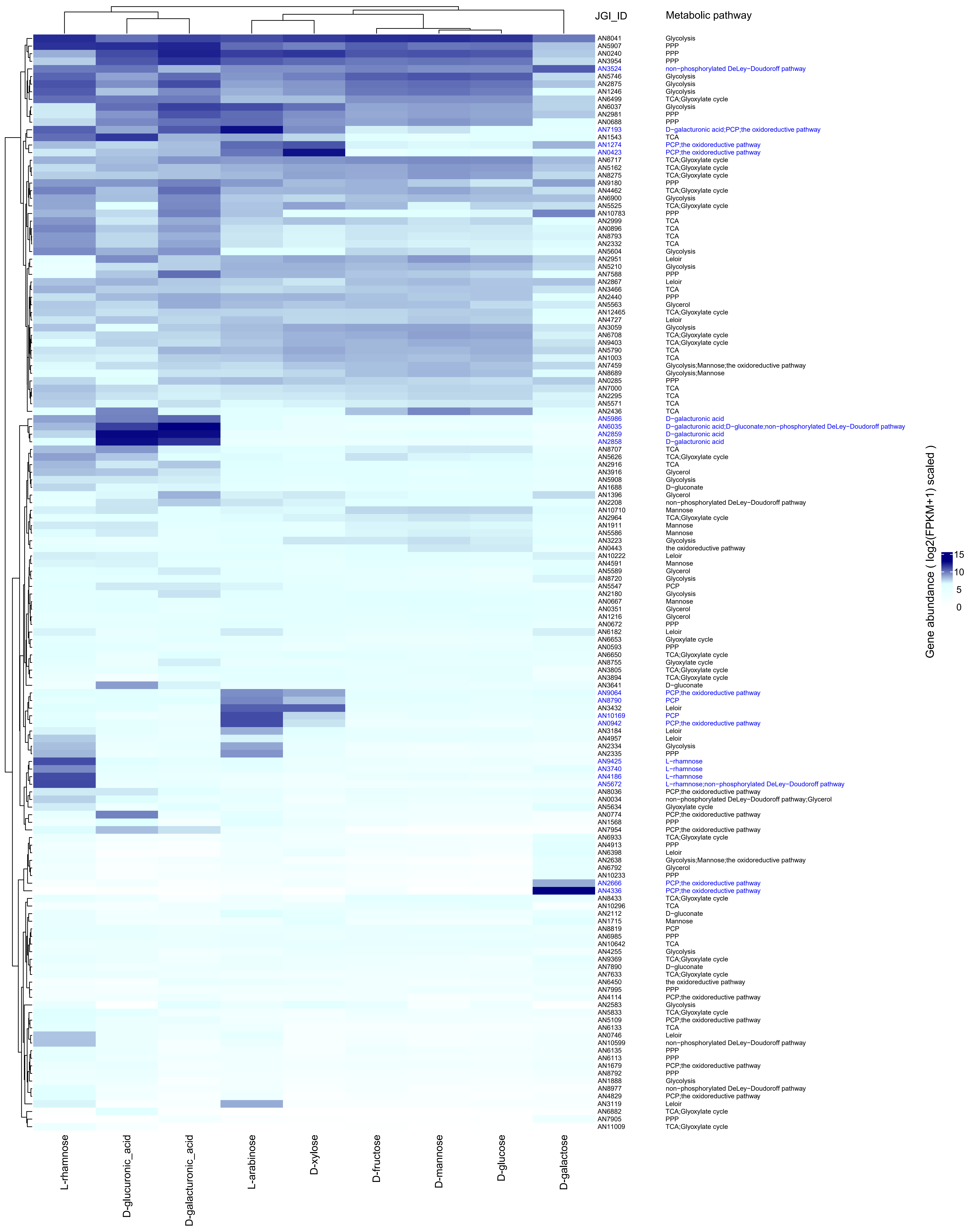

Supplementary Figure S1. B: Expression profiling (FPKM values) of genes involved in sugar metabolic pathways of *A. nidulans.*

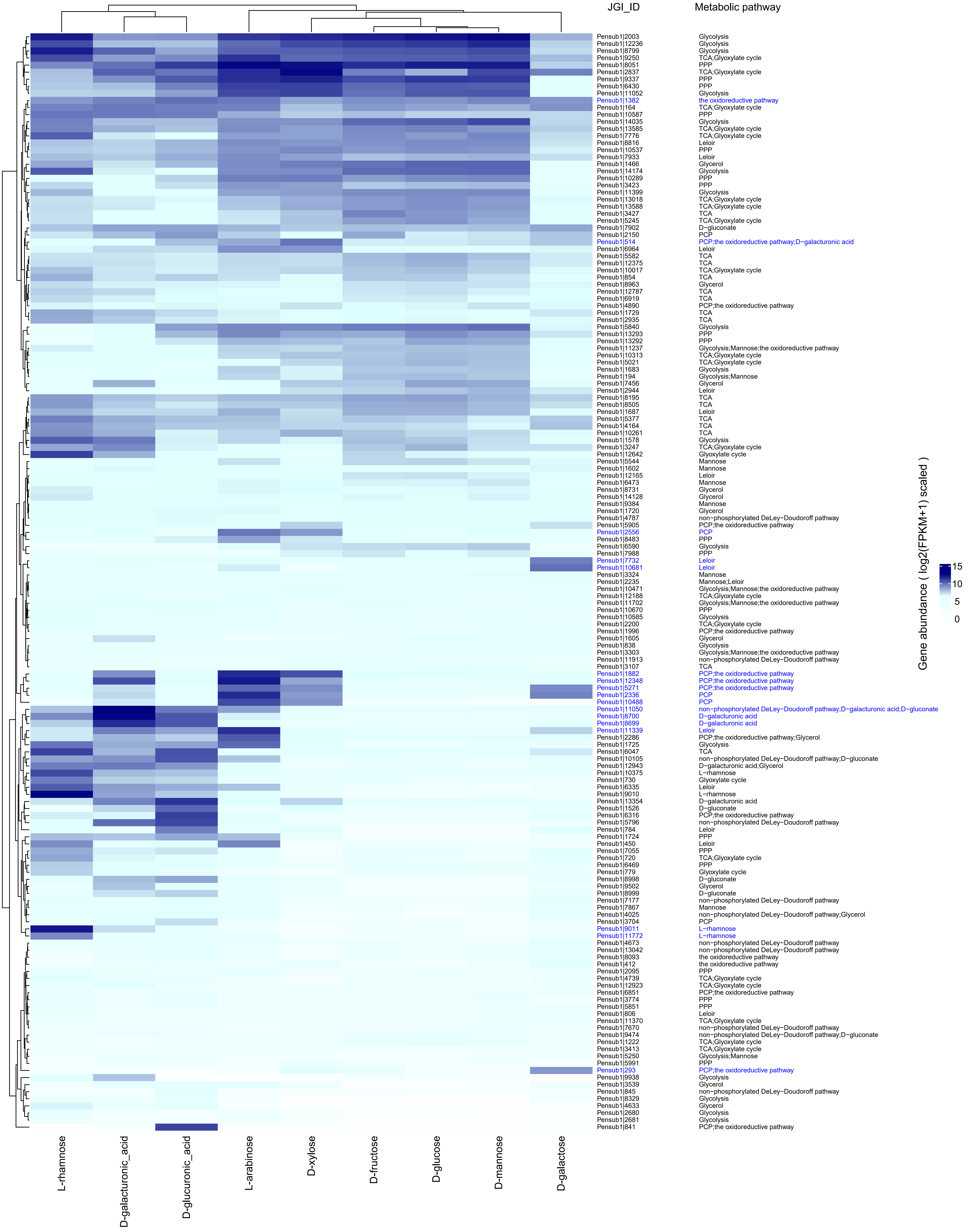

Supplementary Figure S1. C: Expression profiling (FPKM values) of genes involved in sugar metabolic pathways of *P. subrubescens.*

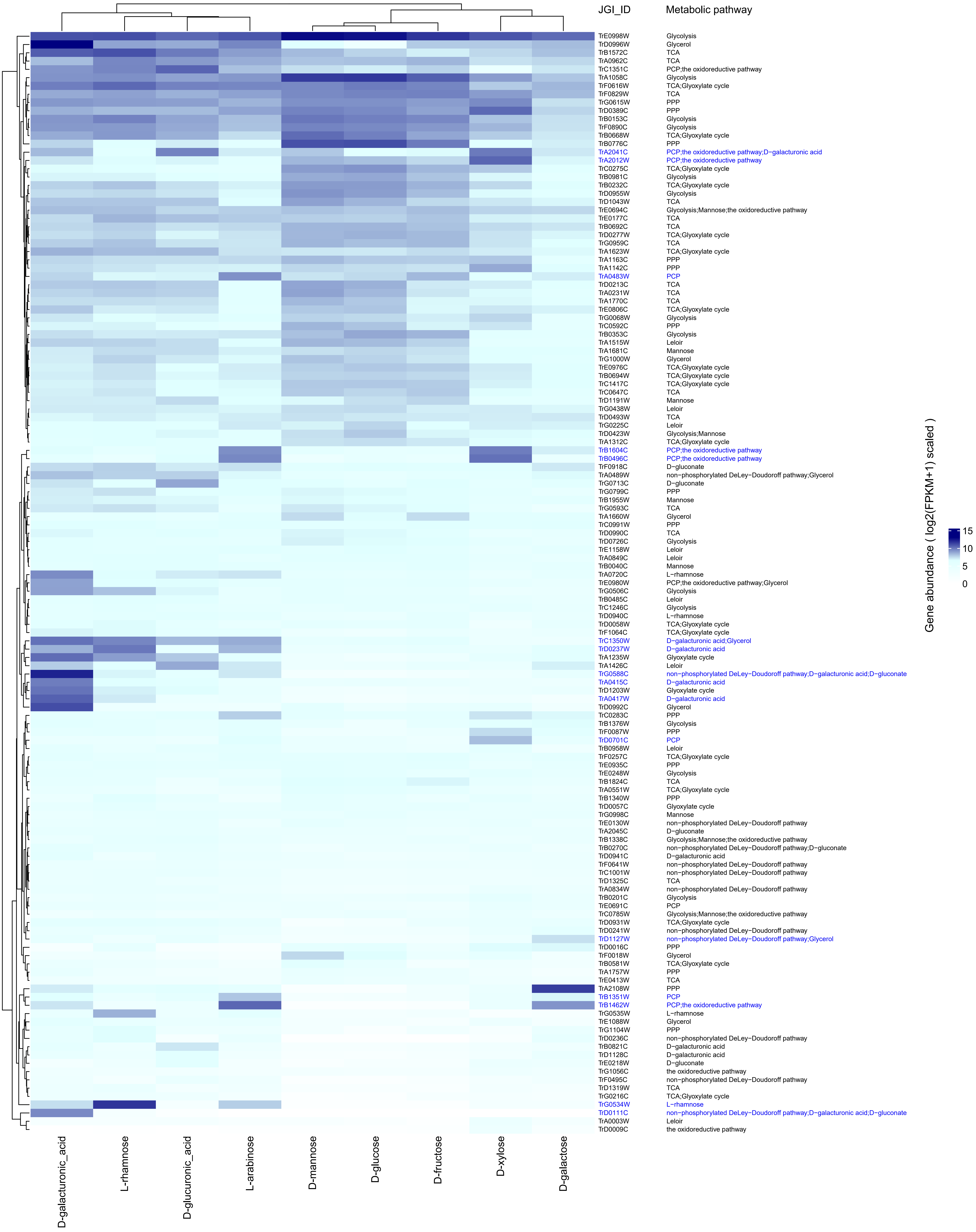

Supplementary Figure S1. D: Expression profiling (FPKM values) of genes involved in sugar metabolic pathways of *T. reesei*.

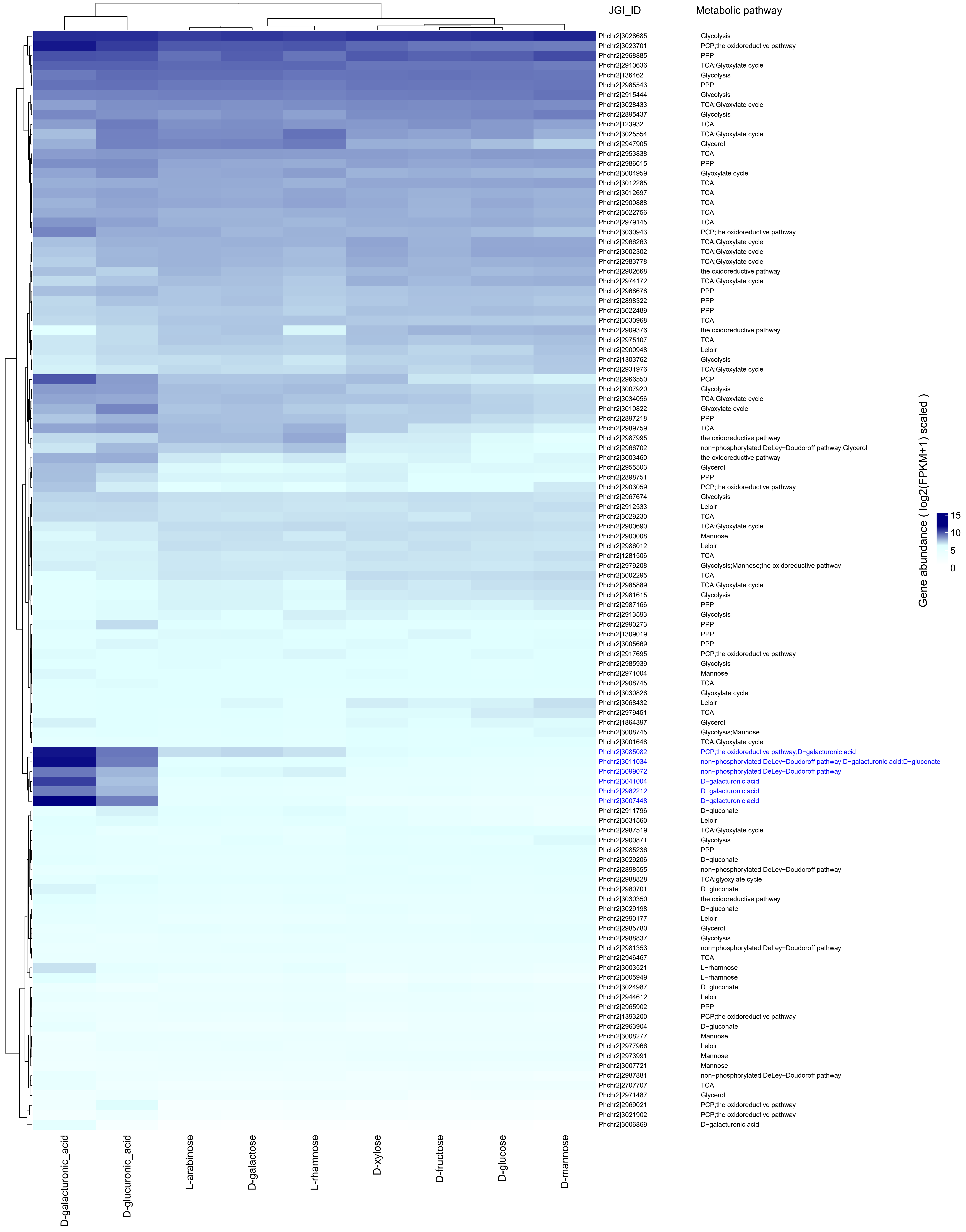

Supplementary Figure S1. E: Expression profiling (FPKM values) of genes involved in sugar metabolic pathways of *P. chrysosporium*.

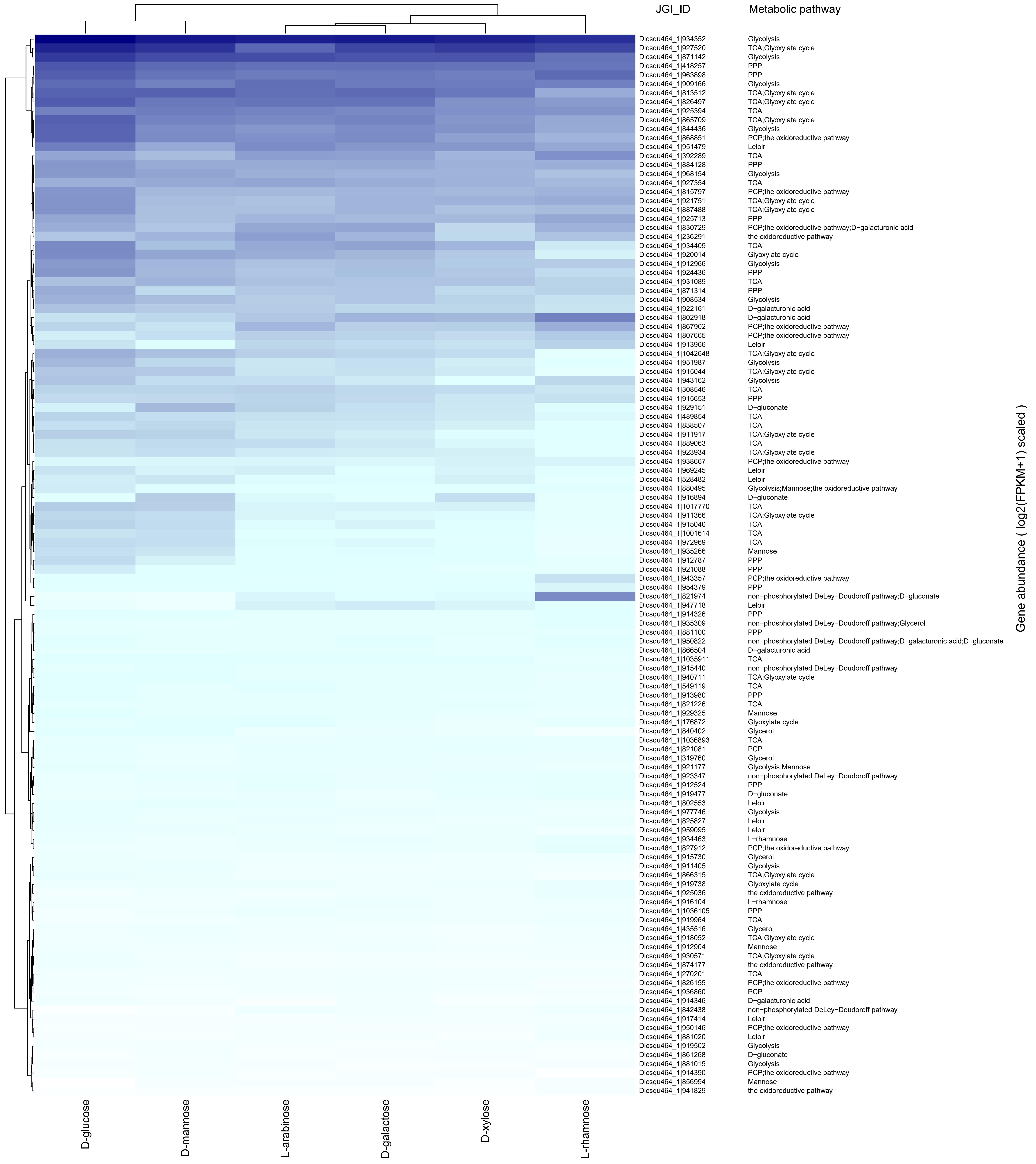

Supplementary Figure S1. F: Expression profiling (FPKM values) of genes involved in sugar metabolic pathways of *D. squalens.*
