# Supplementary material for: The Sugar Metabolic Model of Aspergillus niger Can Only Be Reliably Transferred to Fungi of Its Phylum
Source: J Fungi (Basel). 2022 Dec 17;8(12):1315. doi: 10.3390/jof8121315 (PMC9781776; doi:10.3390/jof8121315)
Supplement: Supplementary file 1 [file jof-08-01315-s001.zip › jof-2056969-supplementary/Supplementary Figure S2.pdf]

Supplementary Figure S2. Heatmap of Pearson correlation between 27 samples of protein abundances (log2 scaled) of all proteins and sugar metabolism-related proteins in five fungi.

Fig. S2. A, **C**, **E**, **G**, **I** indicate correlation plot of protein abundances of all proteins, and **Fig. S2. B**, **D**, **F**, **H**, **J** indicate correlation plot of sugar metabolism-related proteins in *A. niger*, *A. nidulans*, *P. subrubescens*, *T. reesei* and *P. chrysosporium* during their growth on diverse monosaccharides.

The color from blue to dark red indicates a Pearson correlation coefficient from low to high. On the middle bar, different colors indicate different monosaccharides.

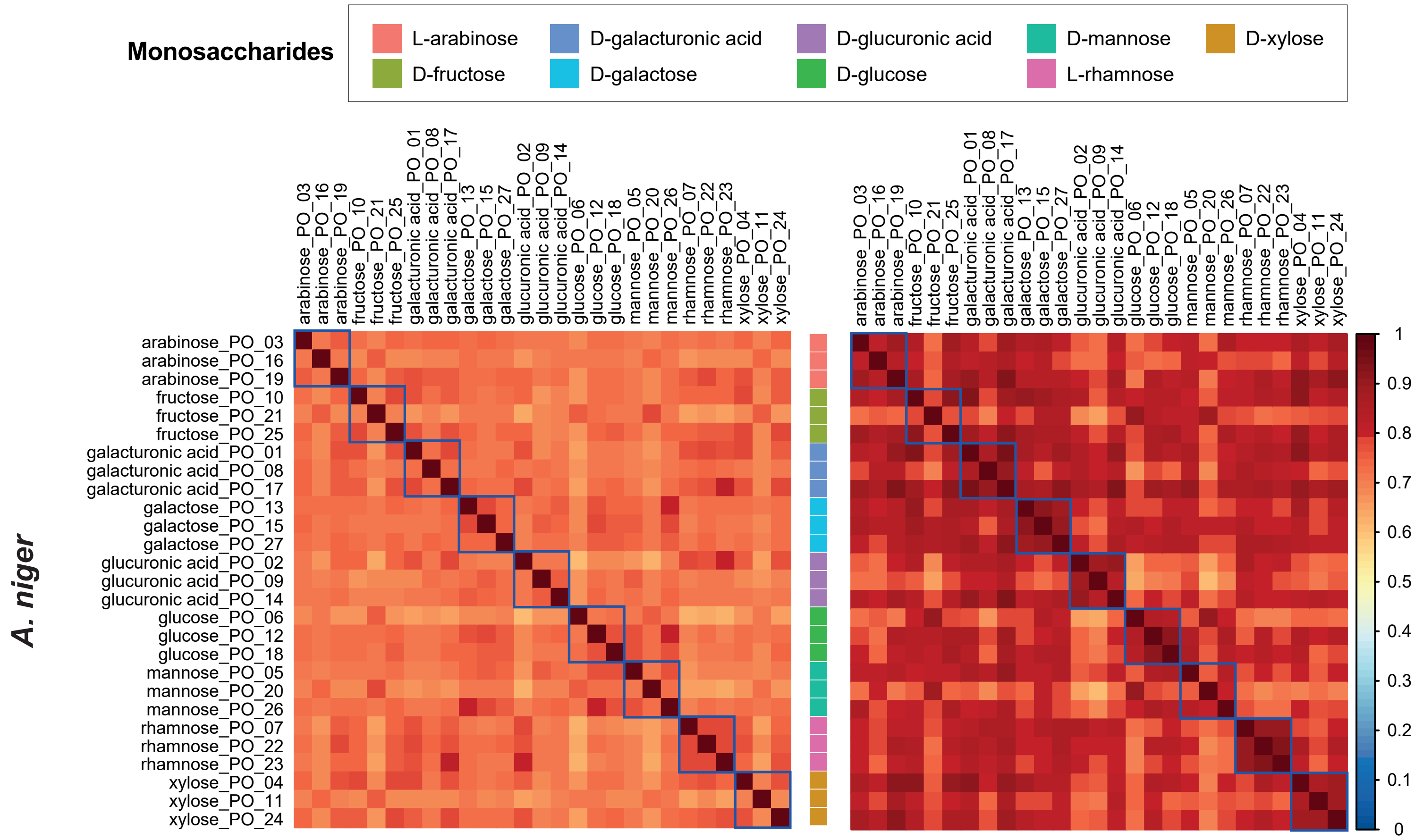

Supplementary Figure S2. A

Supplementary Figure S2. B

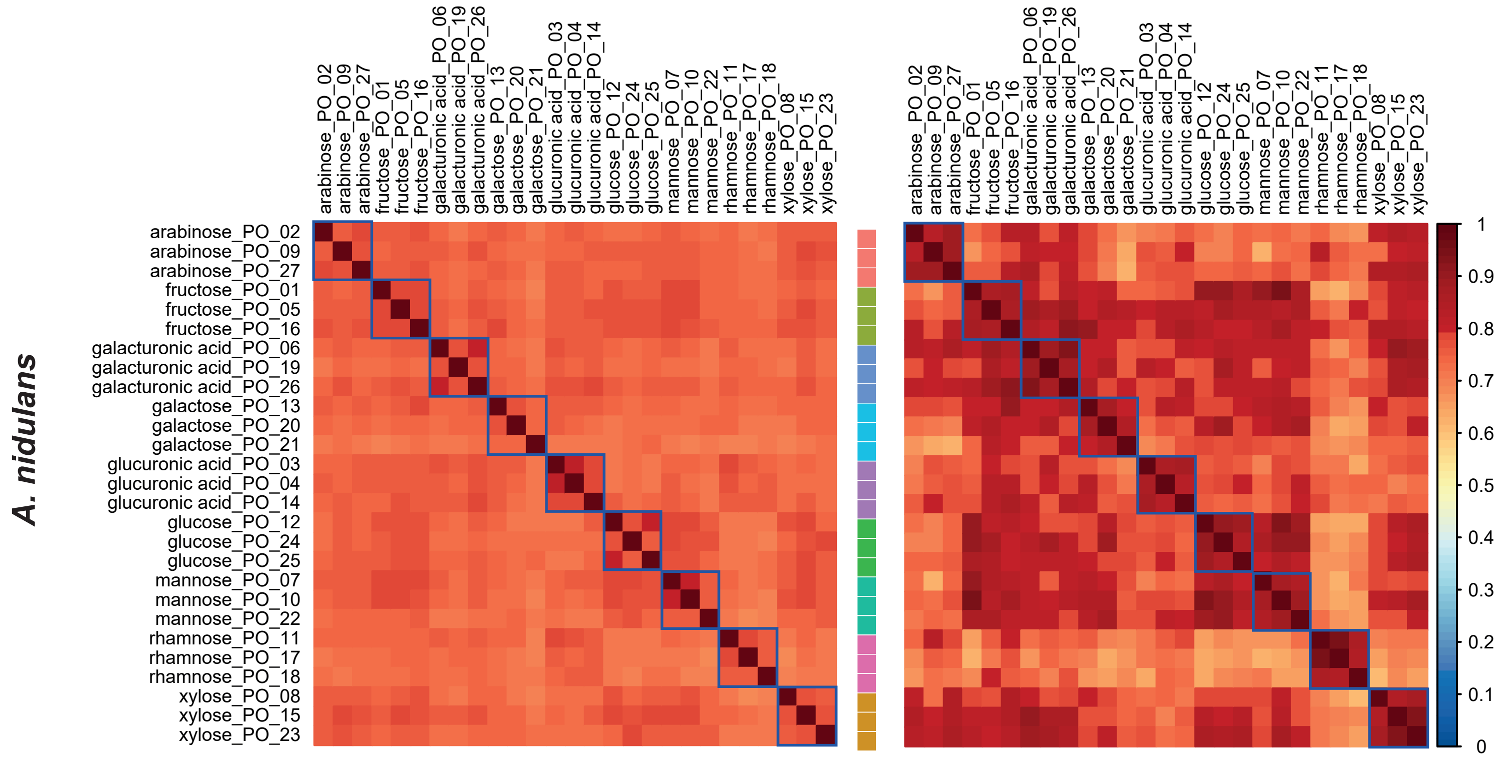

Supplementary Figure S2. C

Supplementary Figure S2. D

P. subrubescens

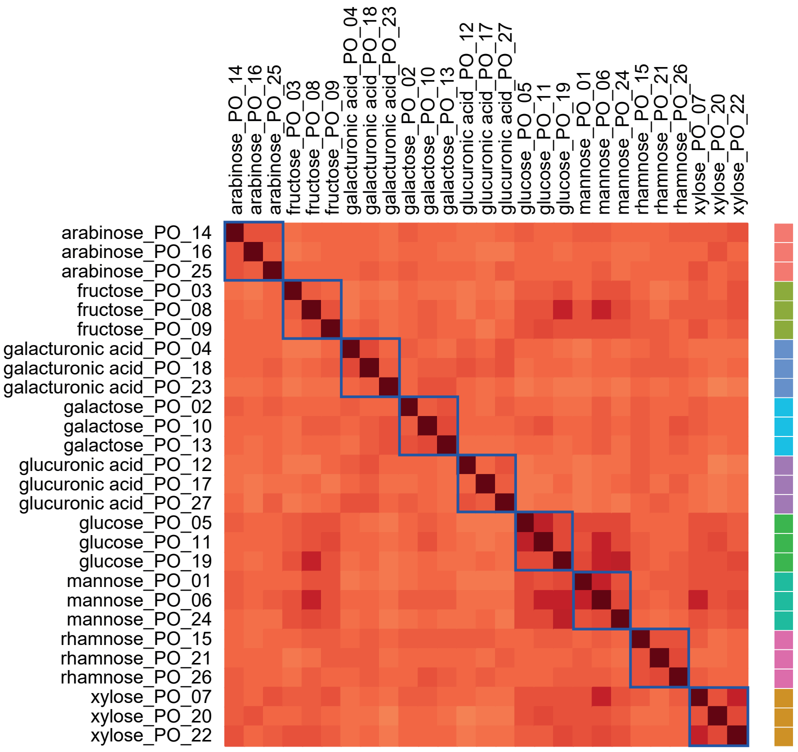

Supplementary Figure S2. E

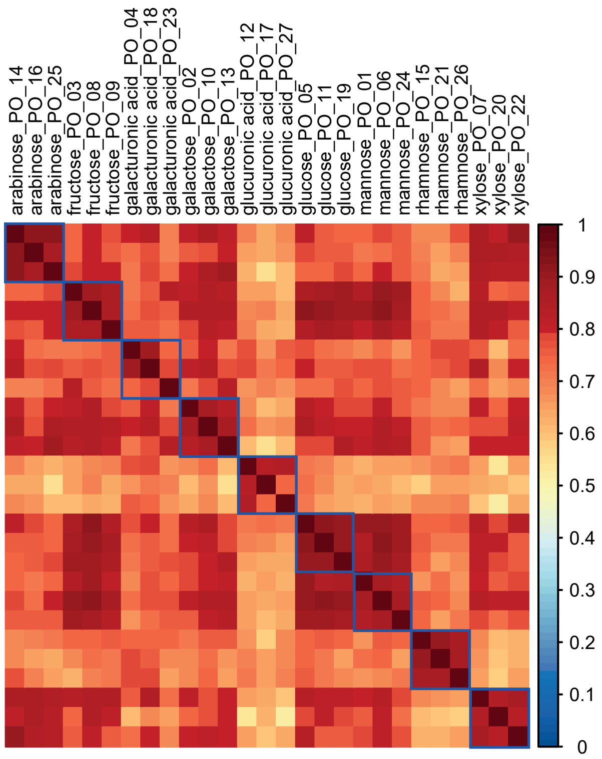

Supplementary Figure S2. F

T. reesei

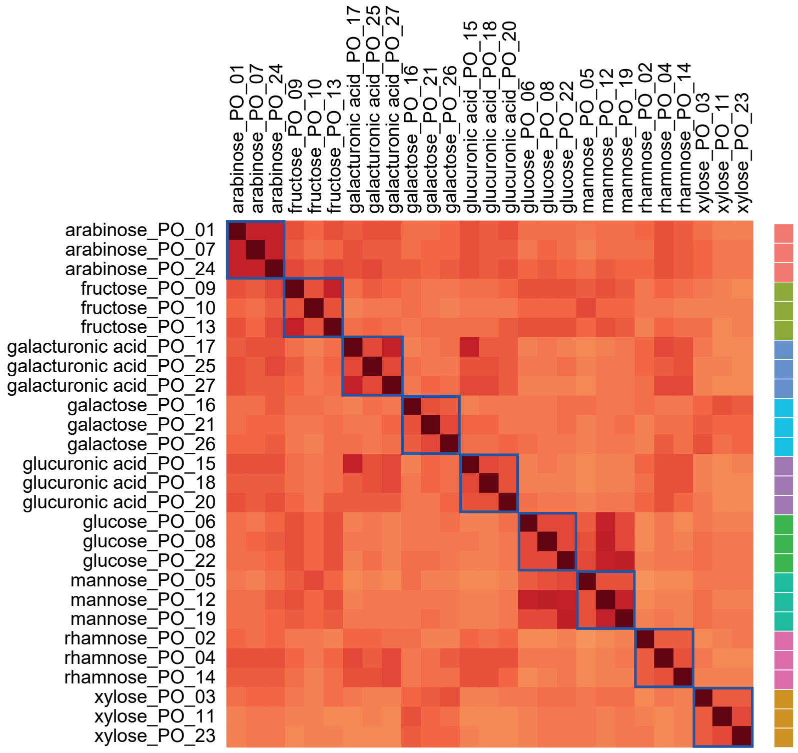

Supplementary Figure S2. G

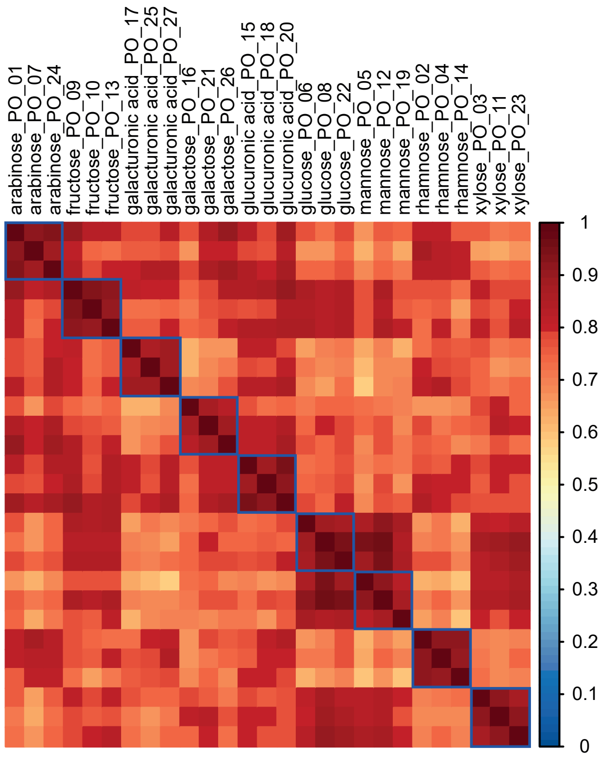

Supplementary Figure S2. H

P. chrysosporium

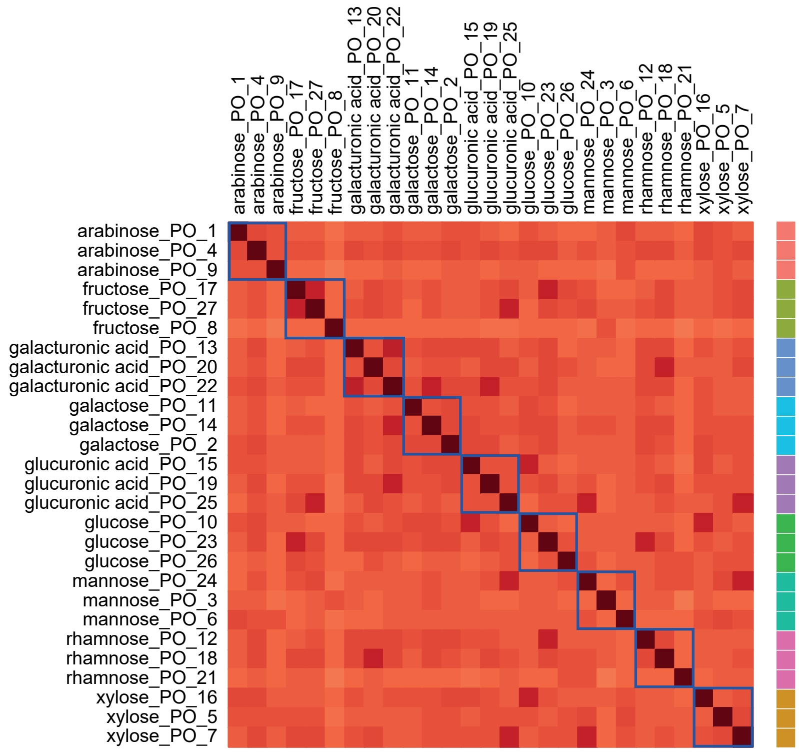

Supplementary Figure S2. I

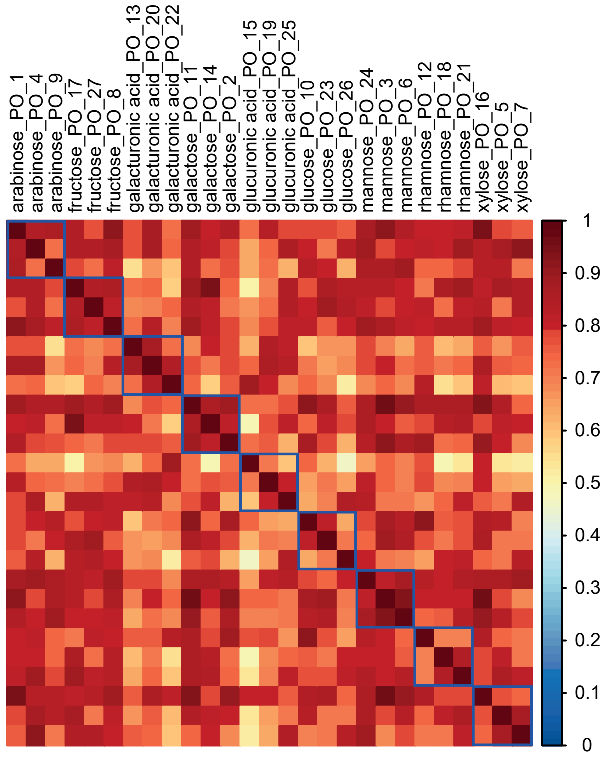

Supplementary Figure S2. J
