# Supplementary material for: The Sugar Metabolic Model of Aspergillus niger Can Only Be Reliably Transferred to Fungi of Its Phylum
Source: J Fungi (Basel). 2022 Dec 17;8(12):1315. doi: 10.3390/jof8121315 (PMC9781776; doi:10.3390/jof8121315)
Supplement: Supplementary file 1 [file jof-08-01315-s001.zip › jof-2056969-supplementary/Supplementary Figure S3.pdf]

Supplementary Figure S3. Protein abundances profile of the sugar metabolism-related proteins involved in each sugar metabolic pathway in *A. niger* (A), *P. subrubescens* (B) and *T. reesei* (C).

Different colors indicate different monosaccharide conditions, and each small circle indicates an individual protein related to each specific sugar metabolic pathway.

The y-axis represents the abundance of proteins (log2 scaled), and the x-axis depicts different sugar metabolic pathways.

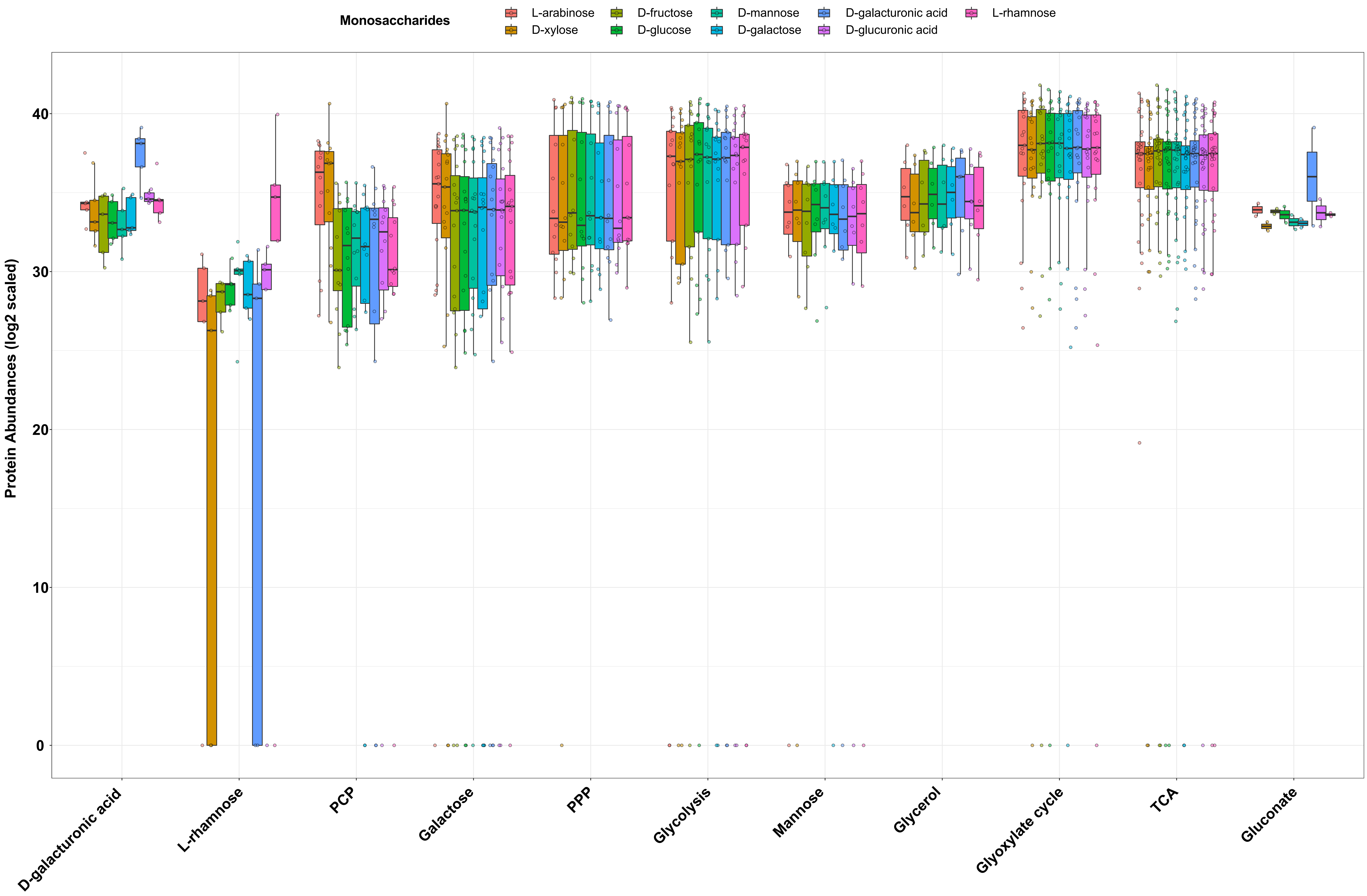

Supplementary Figure S3. A: Protein abundances profile of the sugar metabolism-related proteins in ***A. niger***.

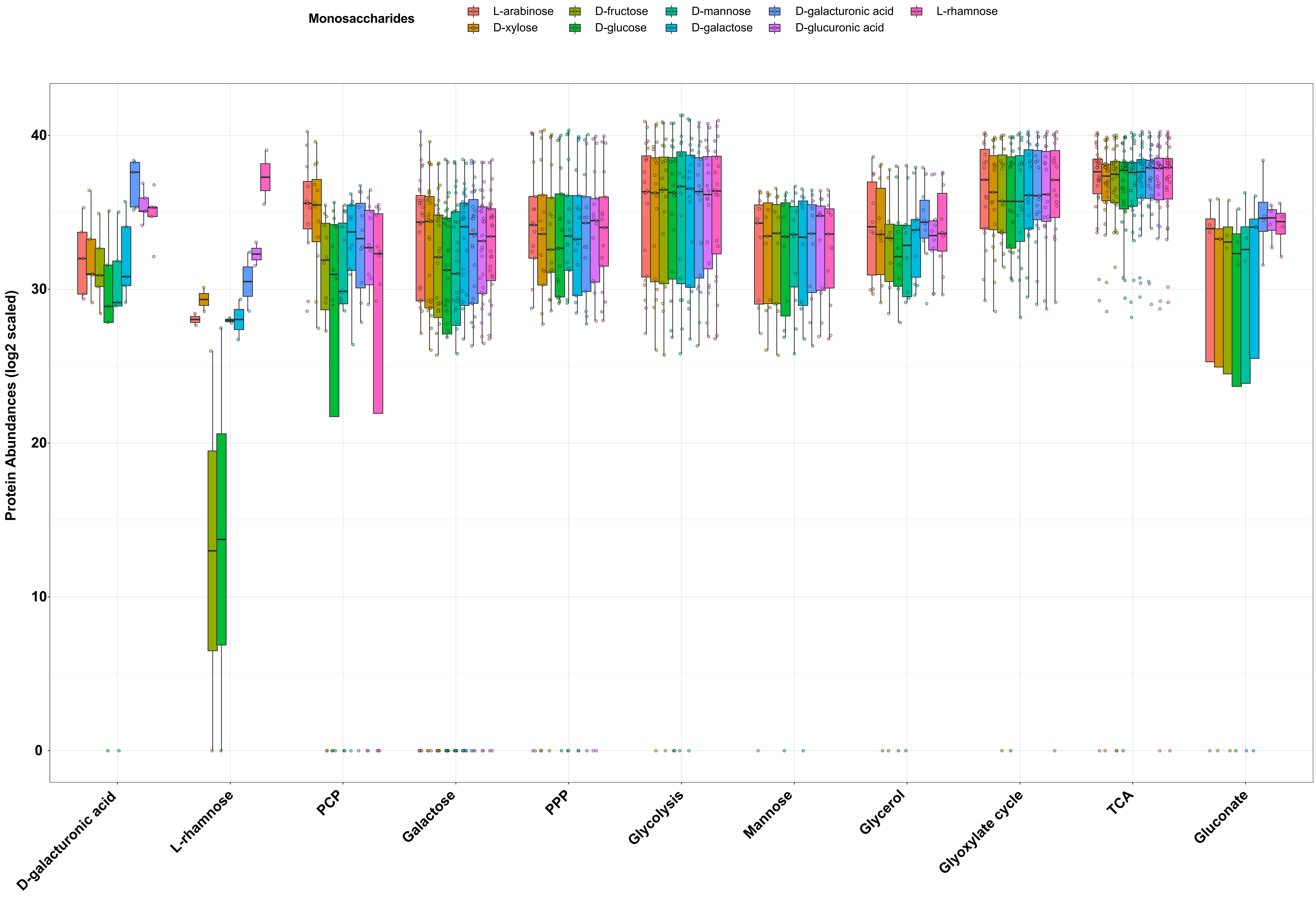

Supplementary Figure S3. B: Protein abundances profile of the sugar metabolism-related proteins in ***P. subrubescens***.

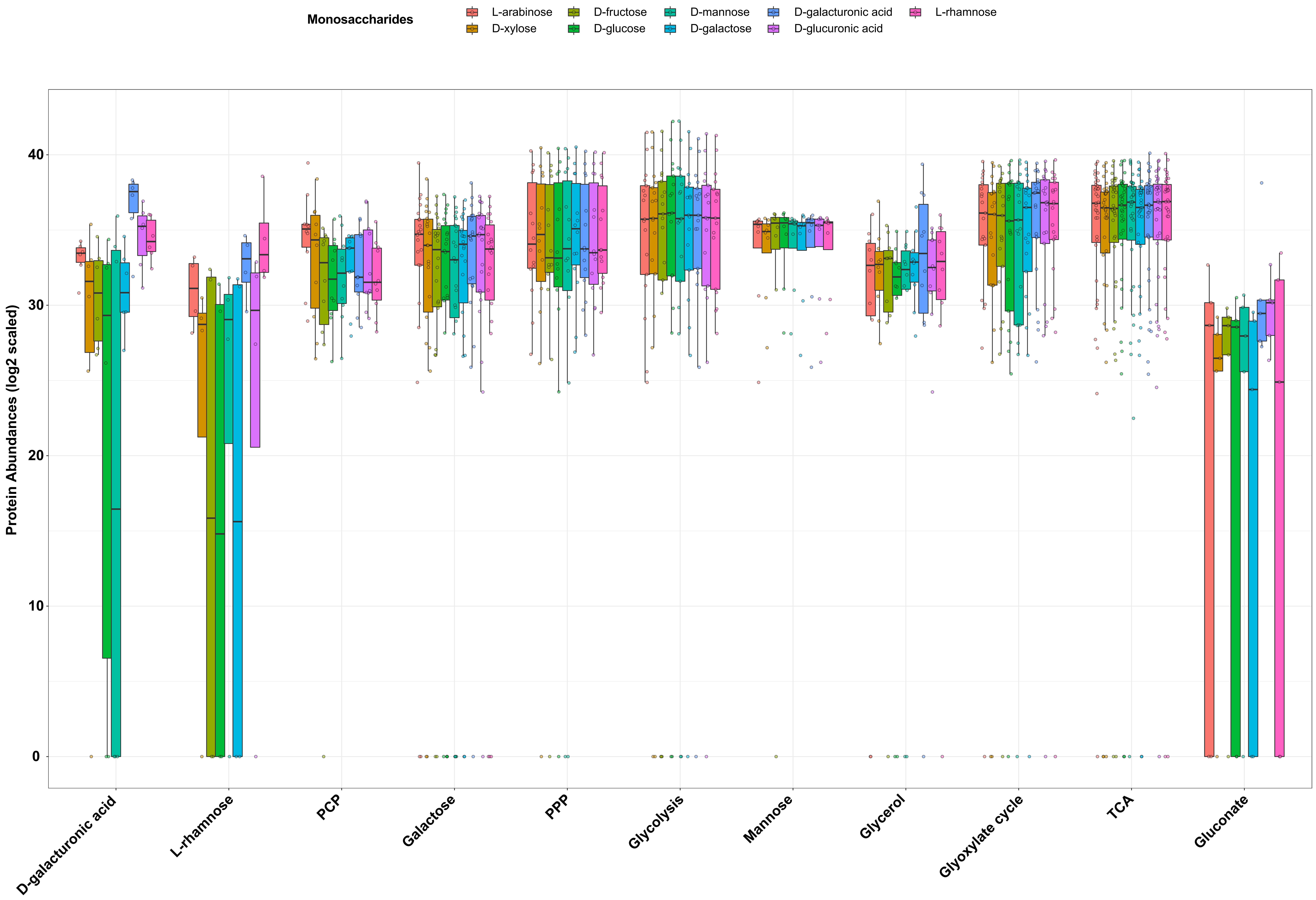

Supplementary Figure S3. C: Protein abundances profile of the sugar metabolism-related proteins in ***T. reesei***.
