# Supplementary material for: The Sugar Metabolic Model of Aspergillus niger Can Only Be Reliably Transferred to Fungi of Its Phylum
Source: J Fungi (Basel). 2022 Dec 17;8(12):1315. doi: 10.3390/jof8121315 (PMC9781776; doi:10.3390/jof8121315)
Supplement: Supplementary file 1 [file jof-08-01315-s001.zip › jof-2056969-supplementary/Supplementary Figure S4.pdf]

Supplementary Figure S4. Heatmap of metabolites abundance levels (normalized log10(abundance levels +1)) of different monosaccharides in *A. niger* (A), *A. nidulans* (B), *P. subrubescens* (C), *T. reesei* (D), and *P. chrysosporium* (E).
The color from blue to red indicates a metabolite abundance level from low to high.

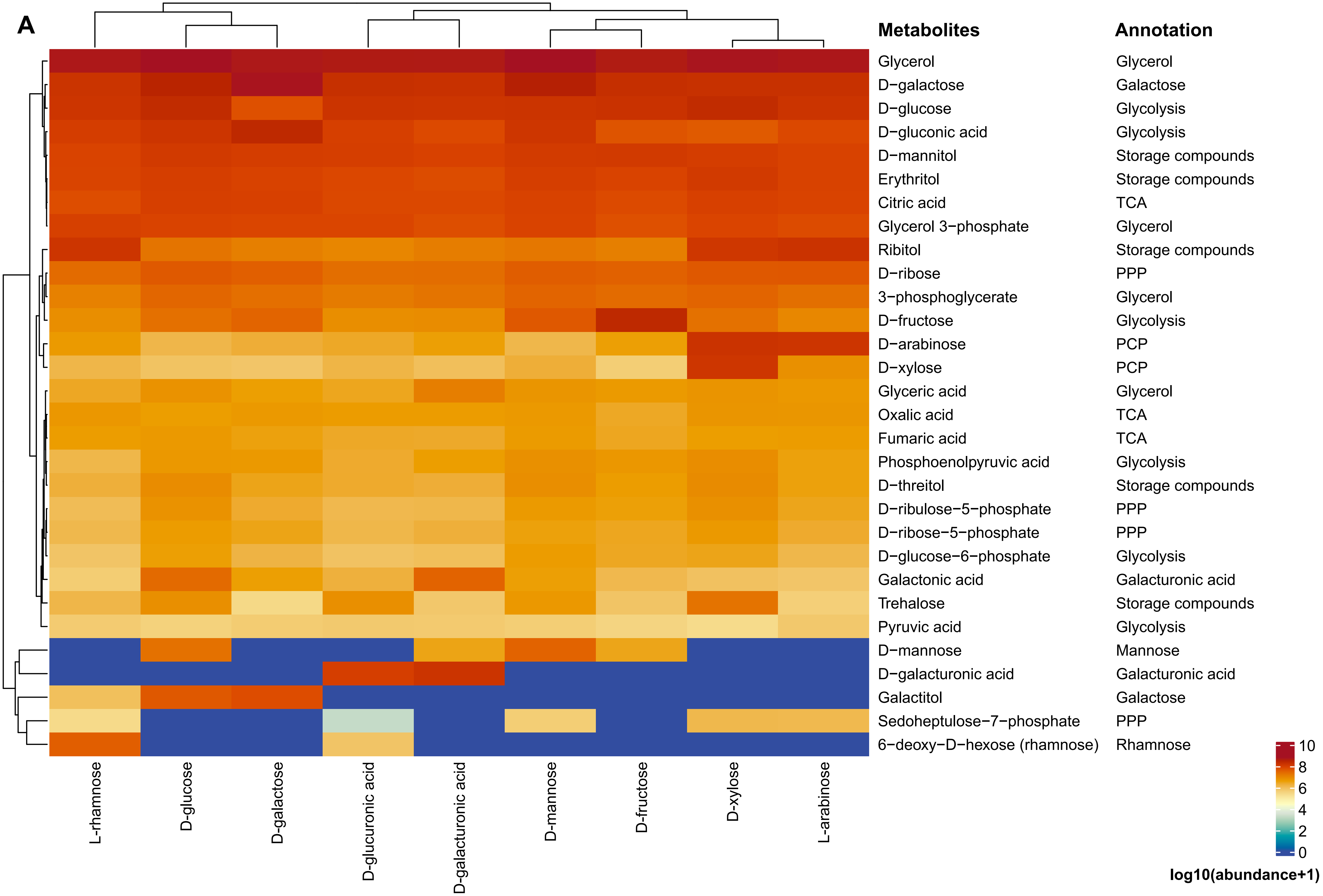

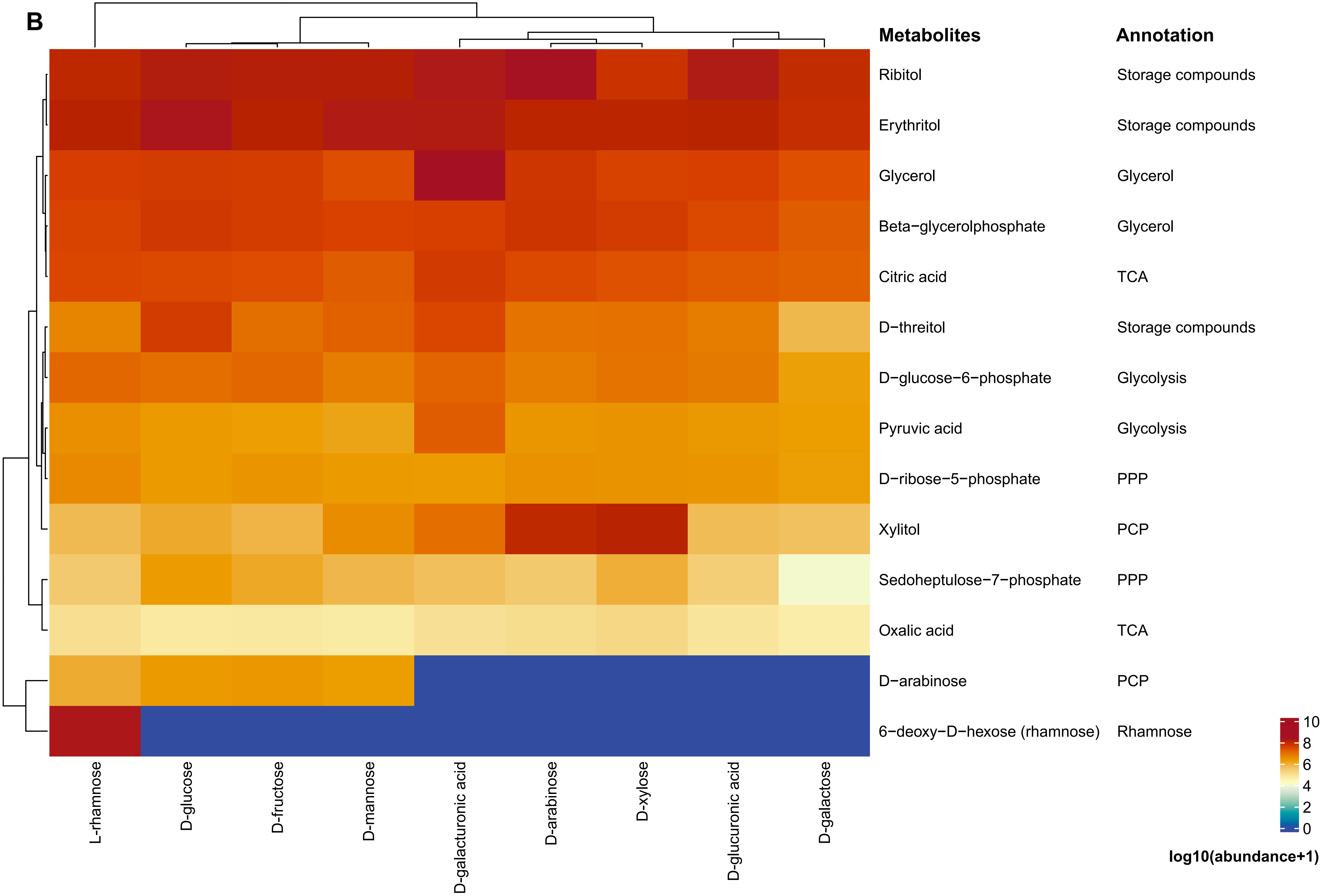
B
Metabolites
Annotation
Ribitol
Storage compounds
Erythritol
Storage compounds
Glycerol
Glycerol
Beta−glycerolphosphate
Glycerol
Citric acid
TCA
D−threitol
Storage compounds
D−glucose−6−phosphate
Glycolysis
Pyruvic acid
Glycolysis
D−ribose−5−phosphate
PPP
Xylitol
PCP
Sedoheptulose−7−phosphate
PPP
Oxalic acid
TCA
D−arabinose
PCP
6−deoxy−D−hexose (rhamnose)
Rhamnose
L-rhamnose
D-glucose
D-fructose
D-mannose
D-galacturonic acid
D-arabinose
D-xylose
D-glucuronic acid
D-galactose
10
8
6
4
2
0
log10(abundance+1)

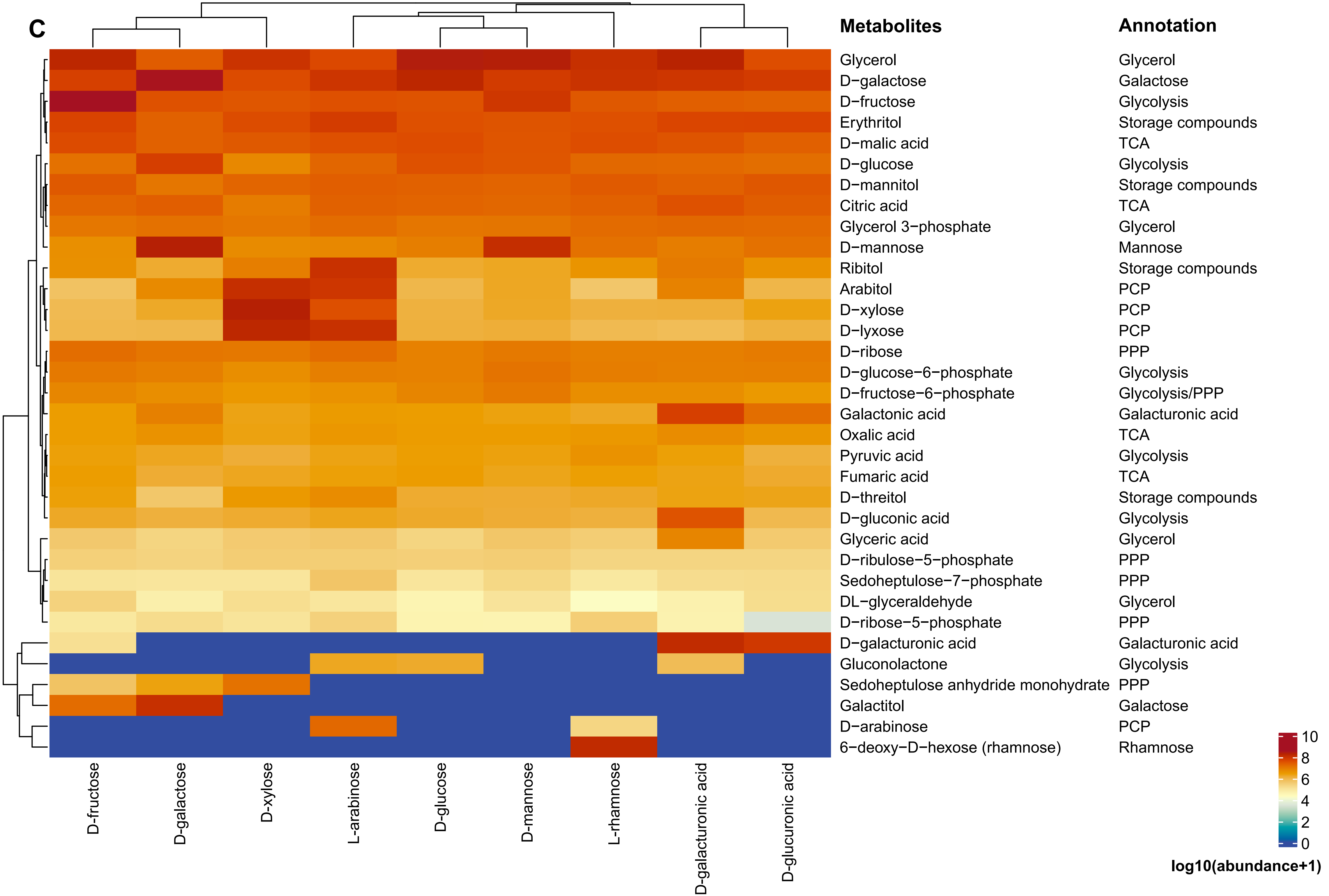
C
Metabolites
Annotation
Glycerol
D−galactose
D−fructose
Erythritol
D−malic acid
D−glucose
D−mannitol
Citric acid
Glycerol 3−phosphate
D−mannose
Ribitol
Arabitol
D−xylose
D−lyxose
D−ribose
D−glucose−6−phosphate
D−fructose−6−phosphate
Galactonic acid
Oxalic acid
Pyruvic acid
Fumaric acid
D−threitol
D−gluconic acid
Glyceric acid
D−ribulose−5−phosphate
Sedoheptulose−7−phosphate
DL−glyceraldehyde
D−ribose−5−phosphate
D−galacturonic acid
Gluconolactone
Sedoheptulose anhydride monohydrate
Galactitol
D−arabinose
6−deoxy−D−hexose (rhamnose)
Glycerol
Galactose
Glycolysis
Storage compounds
TCA
Glycolysis
Storage compounds
TCA
Glycerol
Mannose
Storage compounds
PCP
PCP
PCP
PPP
Glycolysis
Glycolysis/PPP
Galacturonic acid
TCA
Glycolysis
TCA
Storage compounds
Glycolysis
Glycerol
PPP
PPP
Glycerol
PPP
Galacturonic acid
Glycolysis
PPP
Galactose
PCP
Rhamnose
D-fructose
D-galactose
D-xylose
L-arabinose
D-glucose
D-mannose
L-rhamnose
D-galacturonic acid
D-glucuronic acid
10
8
6
4
2
0
log10(abundance+1)

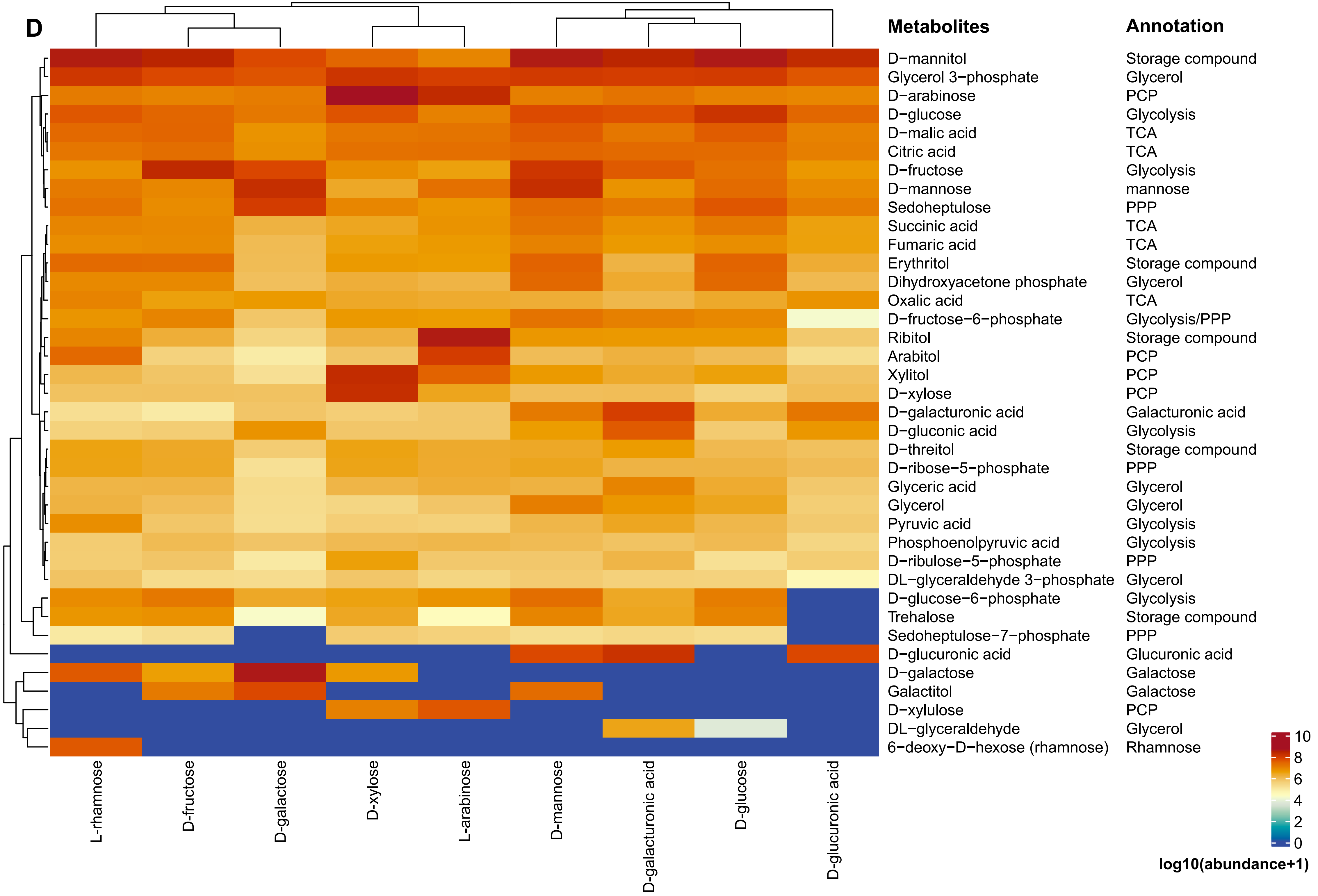

D
Metabolites
Annotation
D−mannitol
Storage compound
Glycerol 3−phosphate
Glycerol
D−arabinose
PCP
D−glucose
Glycolysis
D−malic acid
TCA
Citric acid
TCA
D−fructose
Glycolysis
D−mannose
mannose
Sedoheptulose
PPP
Succinic acid
TCA
Fumaric acid
TCA
Erythritol
Storage compound
Dihydroxyacetone phosphate
Glycerol
Oxalic acid
TCA
D−fructose−6−phosphate
Glycolysis/PPP
Ribitol
Storage compound
Arabitol
PCP
Xylitol
PCP
D−xylose
PCP
D−galacturonic acid
Galacturonic acid
D−gluconic acid
Glycolysis
D−threitol
Storage compound
D−ribose−5−phosphate
PPP
Glyceric acid
Glycerol
Glycerol
Glycerol
Pyruvic acid
Glycolysis
Phosphoenolpyruvic acid
Glycolysis
D−ribulose−5−phosphate
PPP
DL−glyceraldehyde 3−phosphate
Glycerol
D−glucose−6−phosphate
Glycolysis
Trehalose
Storage compound
Sedoheptulose−7−phosphate
PPP
D−glucuronic acid
Glucuronic acid
D−galactose
Galactose
Galactitol
Galactose
D−xylulose
PCP
DL−glyceraldehyde
Glycerol
6−deoxy−D−hexose (rhamnose)
Rhamnose
L-rhamnose
D-fructose
D-galactose
D-xylose
L-arabinose
D-mannose
D-galacturonic acid
D-glucose
D-glucuronic acid
10
8
6
4
2
0
log10(abundance+1)

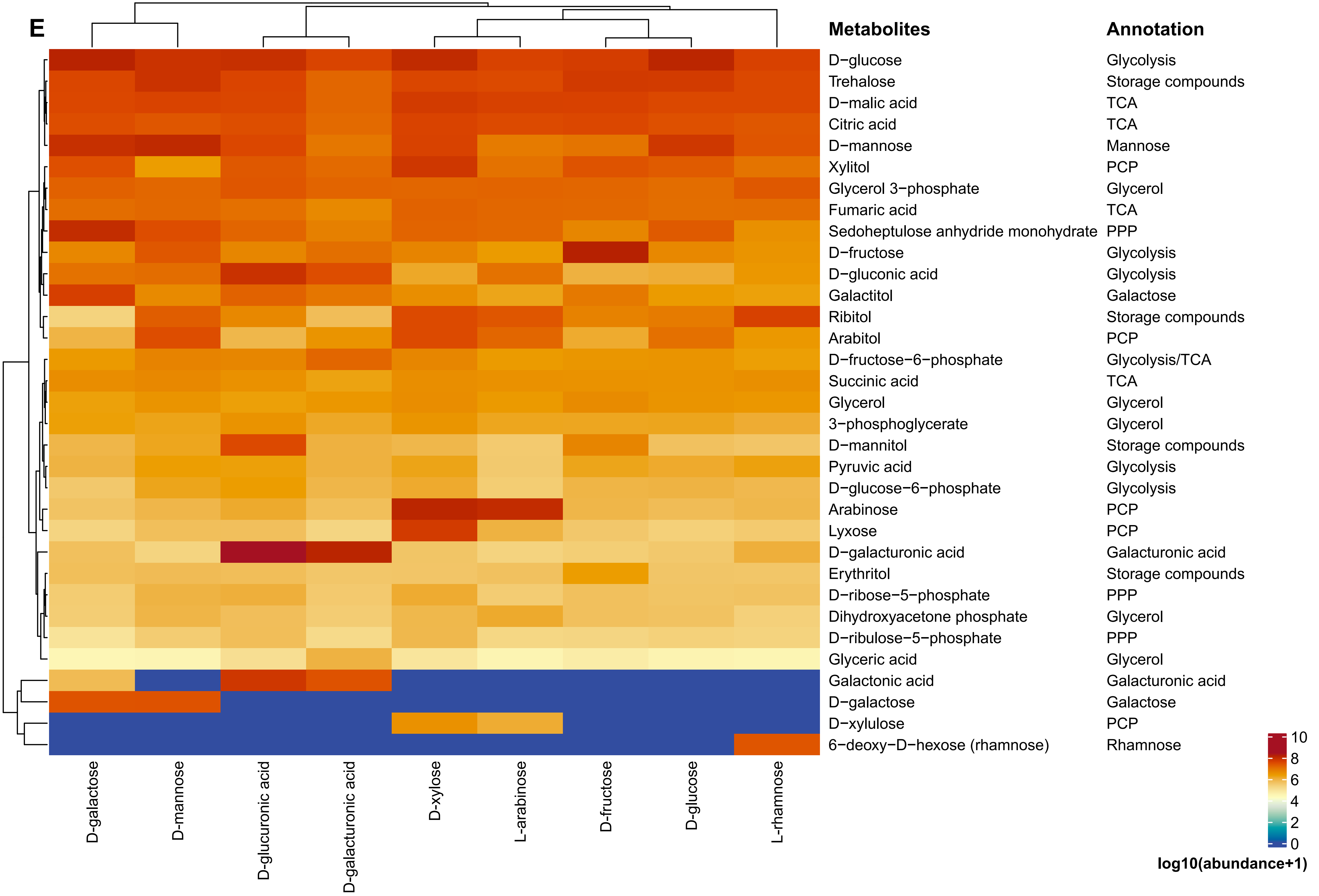
E
Metabolites
Annotation
D−glucose
Glycolysis
Trehalose
Storage compounds
D−malic acid
TCA
Citric acid
TCA
D−mannose
Mannose
Xylitol
PCP
Glycerol 3−phosphate
Glycerol
Fumaric acid
TCA
Sedoheptulose anhydride monohydrate
PPP
D−fructose
Glycolysis
D−gluconic acid
Glycolysis
Galactitol
Galactose
Ribitol
Storage compounds
Arabitol
PCP
D−fructose−6−phosphate
Glycolysis/TCA
Succinic acid
TCA
Glycerol
Glycerol
3−phosphoglycerate
Glycerol
D−mannitol
Storage compounds
Pyruvic acid
Glycolysis
D−glucose−6−phosphate
Glycolysis
Arabinose
PCP
Lyxose
PCP
D−galacturonic acid
Galacturonic acid
Erythritol
Storage compounds
D−ribose−5−phosphate
PPP
Dihydroxyacetone phosphate
Glycerol
D−ribulose−5−phosphate
PPP
Glyceric acid
Glycerol
Galactonic acid
Galacturonic acid
D−galactose
Galactose
D−xylulose
PCP
6−deoxy−D−hexose (rhamnose)
Rhamnose
D-galactose
D-mannose
D-glucuronic acid
D-galacturonic acid
D-xylose
L-arabinose
D-fructose
D-glucose
L-rhamnose
10
8
6
4
2
0
log10(abundance+1)
