# Supplementary material for: The Sugar Metabolic Model of Aspergillus niger Can Only Be Reliably Transferred to Fungi of Its Phylum
Source: J Fungi (Basel). 2022 Dec 17;8(12):1315. doi: 10.3390/jof8121315 (PMC9781776; doi:10.3390/jof8121315)
Supplement: Supplementary file 1 [file jof-08-01315-s001.zip › jof-2056969-supplementary/Supplementary Figure S5.pdf]

Supplementary Figure S5. Correlation between abundance of metabolites and sugar metabolism-related genes in *A. niger* (**A**), *P. subrubescens* (**B**), and *T. reesei* (**C**). The y-axis represents Pearson correlation coefficient (PCC ≥ 0.5), and x-axis depicts different metabolites and the related sugar metabolic pathways (highlighted in blue). Each dot represents a gene and its color indicates the corresponding sugar pathway that it is involved in. The positive and negative correlation are shown in filled and open circles, respectively. Only the names of genes with high correlation (PCC ≥ 0.8) to the analyzed metabolites are displayed.

A

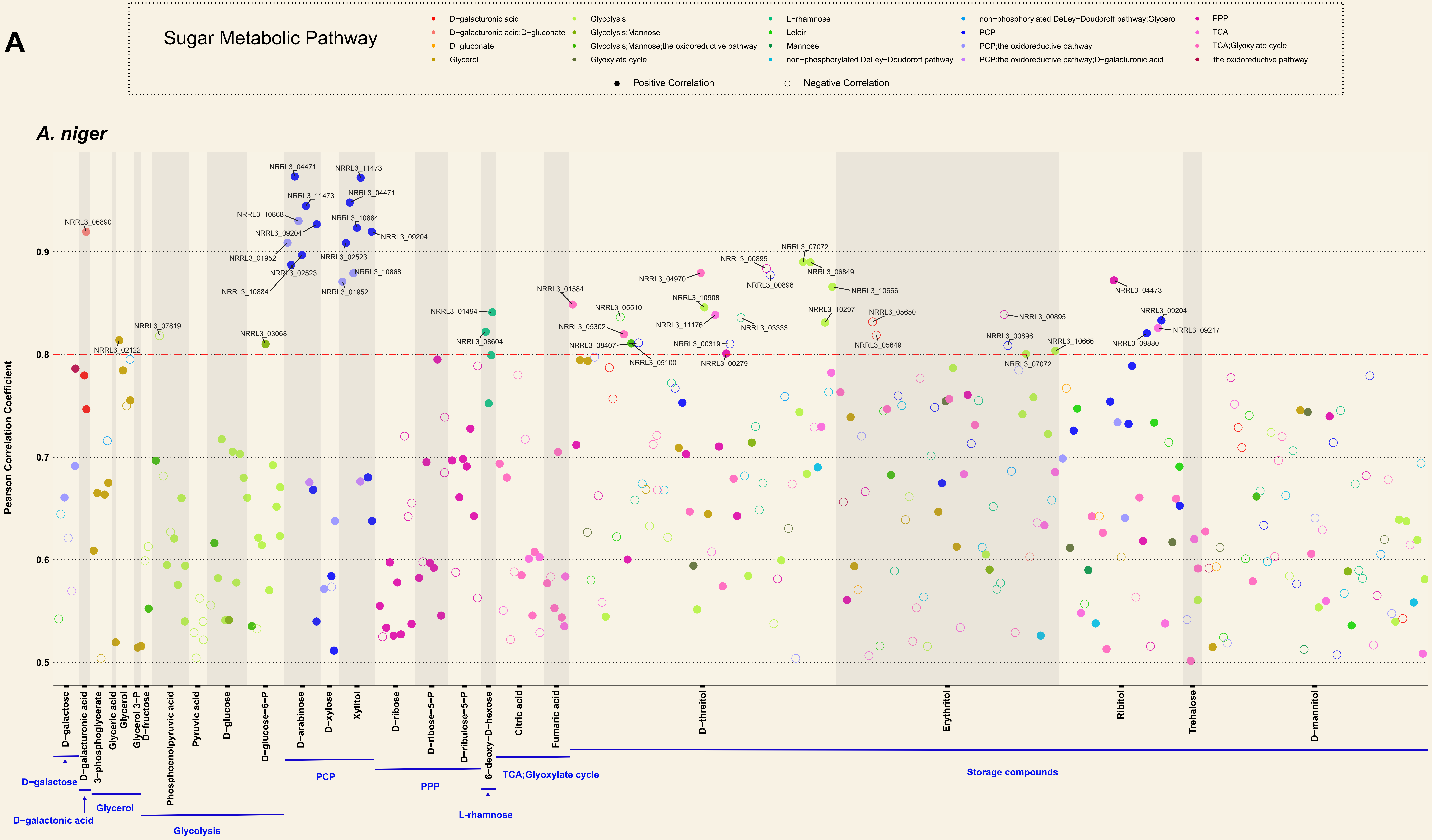

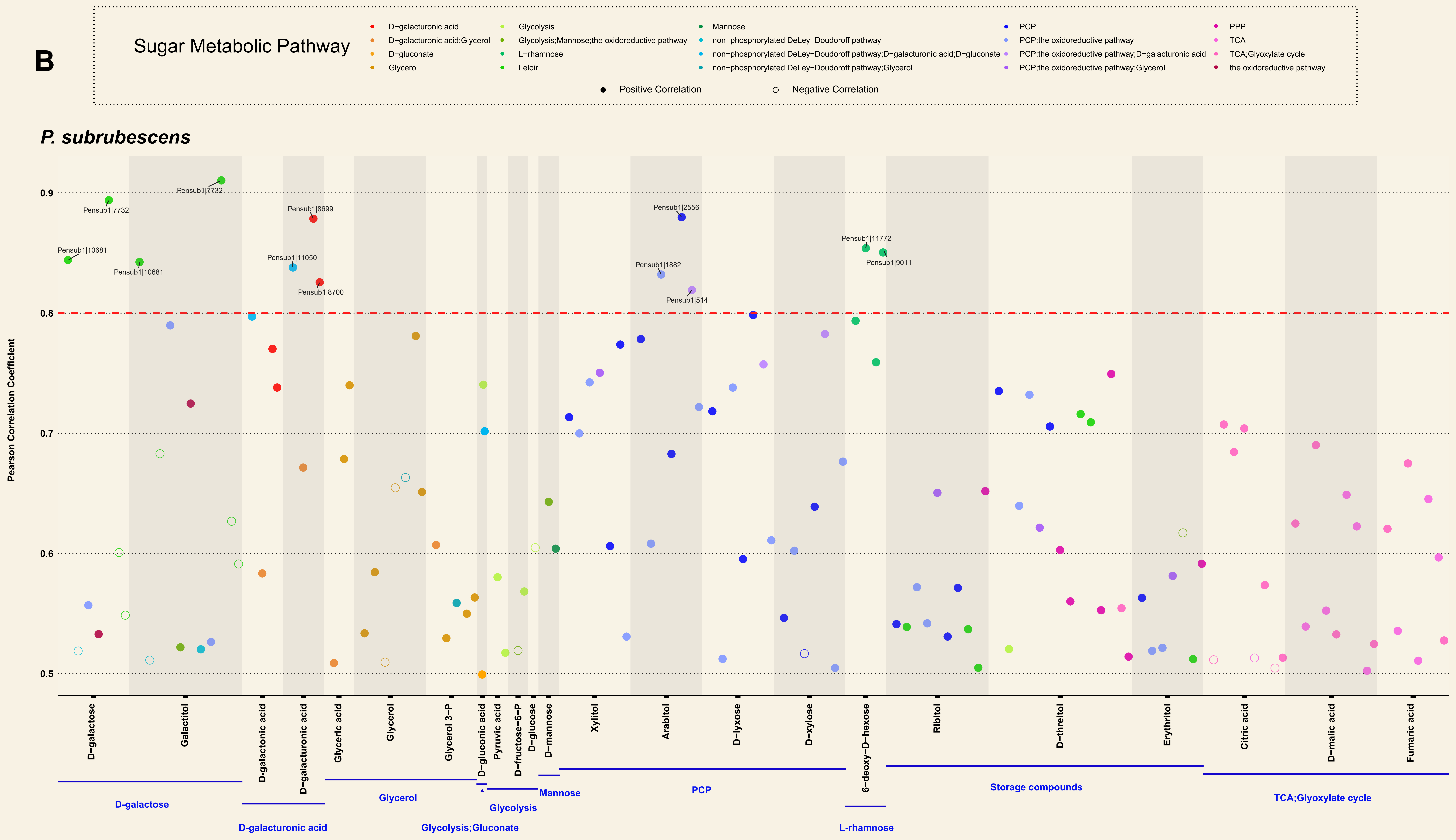

B
Sugar Metabolic Pathway
D−galacturonic acid
D−galacturonic acid;Glycerol
D−gluconate
Glycerol
Glycolysis
Glycolysis;Mannose;the oxidoreductive pathway
L−rhamnose
Leloir
Mannose
non−phosphorylated DeLey−Doudoroff pathway
non−phosphorylated DeLey−Doudoroff pathway;D−galacturonic acid;D−gluconate
non−phosphorylated DeLey−Doudoroff pathway;Glycerol
PCP
PCP;the oxidoreductive pathway
PCP;the oxidoreductive pathway;D−galacturonic acid
PCP;the oxidoreductive pathway;Glycerol
PPP
TCA
TCA;Glyoxylate cycle
the oxidoreductive pathway
Positive Correlation
Negative Correlation
P. subrubescens
Pearson Correlation Coefficient
0.9
0.8
0.7
0.6
0.5
Pensub1|7732
Pensub1|7732
Pensub1|10681
Pensub1|10681
Pensub1|8699
Pensub1|11050
Pensub1|8700
Pensub1|2556
Pensub1|1882
Pensub1|514
Pensub1|11772
Pensub1|9011
D−galactose
Galactitol
D−galactonic acid
D−galacturonic acid
Glyceric acid
Glycerol
Glycerol 3−P
D−gluconic acid
Pyruvic acid
D−fructose−6−P
D−glucose
D−mannose
Xylitol
Arabitol
D−lyxose
D−xylose
6−deoxy−D−hexose
Ribitol
D−threitol
Erythritol
Citric acid
D−malic acid
Fumaric acid
D-galactose
D-galacturonic acid
Glycerol
Glycolysis;Gluconate
Glycolysis
Mannose
PCP
L-rhamnose
Storage compounds
TCA;Glyoxylate cycle

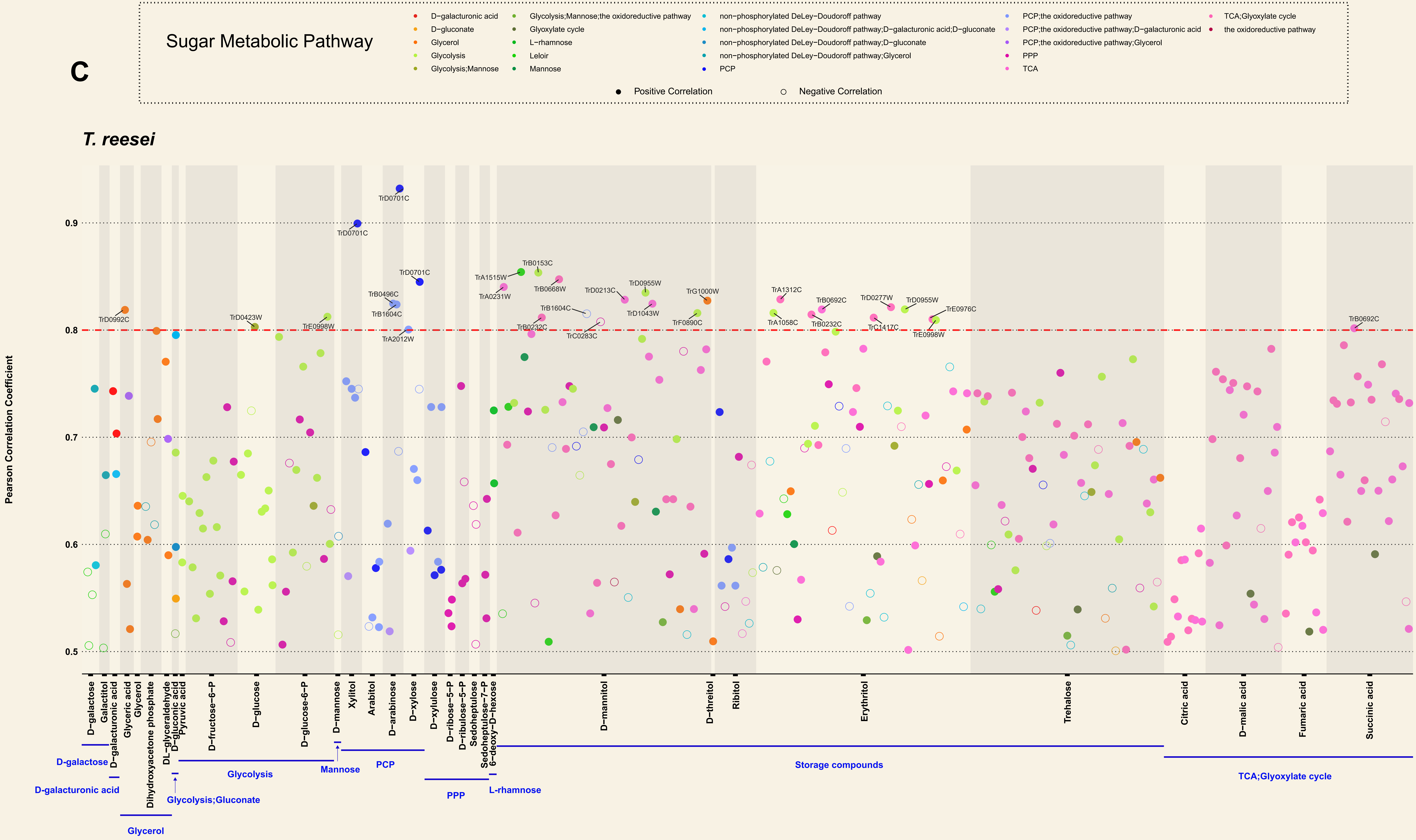
C
Sugar Metabolic Pathway
D−galacturonic acid
D−gluconate
Glycerol
Glycolysis
Glycolysis;Mannose
Glycolysis;Mannose;the oxidoreductive pathway
Glyoxylate cycle
L−rhamnose
Leloir
Mannose
non−phosphorylated DeLey−Doudoroff pathway
non−phosphorylated DeLey−Doudoroff pathway;D−galacturonic acid;D−gluconate
non−phosphorylated DeLey−Doudoroff pathway;D−gluconate
non−phosphorylated DeLey−Doudoroff pathway;Glycerol
PCP
PCP;the oxidoreductive pathway
PCP;the oxidoreductive pathway;D−galacturonic acid
PCP;the oxidoreductive pathway;Glycerol
PPP
TCA
TCA;Glyoxylate cycle
the oxidoreductive pathway
Positive Correlation
Negative Correlation
T. reesei
Pearson Correlation Coefficient
0.9
0.8
0.7
0.6
0.5
D−galactose
Galactitol
D−galacturonic acid
Glyceric acid
Glycerol
Dihydroxyacetone phosphate
DL−glyceraldehyde
D−gluconic acid
Pyruvic acid
D−fructose−6−P
D−glucose
D−glucose−6−P
D−mannose
Xylitol
Arabitol
D−arabinose
D−xylose
D−xylulose
D−ribose−5−P
D−ribulose−5−P
Sedoheptulose
Sedoheptulose−7−P
6−deoxy−D−hexose
D−mannitol
D−threitol
Ribitol
Erythritol
Trehalose
Citric acid
D−malic acid
Fumaric acid
Succinic acid
D-galactose
D-galacturonic acid
Glycerol
Glycolysis
Glycolysis;Gluconate
Mannose
PCP
PPP
L-rhamnose
Storage compounds
TCA;Glyoxylate cycle
