# Supplementary material for: The Sugar Metabolic Model of Aspergillus niger Can Only Be Reliably Transferred to Fungi of Its Phylum
Source: J Fungi (Basel). 2022 Dec 17;8(12):1315. doi: 10.3390/jof8121315 (PMC9781776; doi:10.3390/jof8121315)
Supplement: Supplementary file 1 [file jof-08-01315-s001.zip › jof-2056969-supplementary/Supplementary Table S1.docx]

**Supplementary Table 1**. List of species used in this study.

| Species | Strain | References | Genome URL |
| --- | --- | --- | --- |
| *Aspergillus niger* | NRRL3 | [1,2] | https://mycocosm.jgi.doe.gov/Aspni_NRRL3_1/Aspni_NRRL3_1.home.html |
| *Aspergillus nidulans* | FGSC A4 | [3,4] | https://mycocosm.jgi.doe.gov/Aspnid1/Aspnid1.home.html |
| *Penicillium subrubescens* | FBCC1632/CBS132785 | [5] | https://mycocosm.jgi.doe.gov/Pensub1/Pensub1.home.html |
| *Trichoderma reesei* | QM6a | [6,7] | https://mycocosm.jgi.doe.gov/Trire_Chr/Trire_Chr.home.html |
| *Phanerochaete chrysosporium* | PR-78 | [8] | https://mycocosm.jgi.doe.gov/Phchr2/Phchr2.home.html |
| *Dichomitus squalens* | CBS 464.89 | [9] | https://mycocosm.jgi.doe.gov/Dicsqu464_1/Dicsqu464_1.home.html |

**References**

[1] Vesth TC, Nybo JL, Theobald S, Frisvad JC, Larsen TO, Nielsen KF*, et al.* Investigation of inter-and intraspecies variation through genome sequencing of *Aspergillus* section *Nigri.* Nat Genet 2018;50:1688-95. <https://doi.org/10.1038/s41588-018-0246-1>.

[2] Aguilar-Pontes MV, Brandl J, McDonnell E, Strasser K, Nguyen T, Riley R*, et al.* The gold-standard genome of *Aspergillus niger* NRRL 3 enables a detailed view of the diversity of sugar catabolism in fungi*.* Stud Mycol 2018;91:61-78. <https://doi.org/10.1016/j.simyco.2018.10.001>.

[3] Arnaud MB, Cerqueira GC, Inglis DO, Skrzypek MS, Binkley J, Chibucos MC*, et al.* The *Aspergillus* Genome Database (AspGD): recent developments in comprehensive multispecies curation, comparative genomics and community resources*.* Nucleic Acids Res 2012;40:D653-D9. <https://doi.org/10.1093/nar/gkr875>.

[4] Galagan JE, Calvo SE, Cuomo C, Ma L-J, Wortman JR, Batzoglou S*, et al.* Sequencing of *Aspergillus nidulans* and comparative analysis with *A. fumigatus* and *A. oryzae.* Nature 2005;438:1105-15. <https://doi.org/10.1038/nature04341>.

[5] Peng M, Dilokpimol A, Mäkelä MR, Hildén K, Bervoets S, Riley R*, et al.* The draft genome sequence of the ascomycete fungus *Penicillium subrubescens* reveals a highly enriched content of plant biomass related CAZymes compared to related fungi*.* J Biotechnol 2017;246:1-3. <https://doi.org/10.1016/j.jbiotec.2017.02.012>.

[6] Li W-C, Huang C-H, Chen C-L, Chuang Y-C, Tung S-Y, Wang T-F. *Trichoderma reesei* complete genome sequence, repeat-induced point mutation, and partitioning of CAZyme gene clusters*.* Biotechnol Biofuels 2017;10:1-20. <https://doi.org/10.1186/s13068-017-0825-x>.

[7] Martinez D, Berka RM, Henrissat B, Saloheimo M, Arvas M, Baker SE*, et al.* Genome sequencing and analysis of the biomass-degrading fungus *Trichoderma reesei* (syn. *Hypocrea jecorina*)*.* Nat Biotechnol 2008;26:553-60. <https://doi.org/10.1038/nbt1403>.

[8] Ohm RA, Riley R, Salamov A, Min B, Choi I-G, Grigoriev IV. Genomics of wood-degrading fungi*.* Fungal Genet Biol 2014;72:82-90. <https://doi.org/10.1016/j.fgb.2014.05.001>.

[9] Casado López S, Peng M, Daly P, Andreopoulos B, Pangilinan J, Lipzen A*, et al.* Draft genome sequences of three monokaryotic isolates of the white-rot basidiomycete fungus *Dichomitus squalens.* Microbiol Resour Announc 2019;8:e00264-19. <https://doi.org/10.1128/mra.00264-19>.
