# Supplementary material for: The Sugar Metabolic Model of Aspergillus niger Can Only Be Reliably Transferred to Fungi of Its Phylum
Source: J Fungi (Basel). 2022 Dec 17;8(12):1315. doi: 10.3390/jof8121315 (PMC9781776; doi:10.3390/jof8121315)
Supplement: Supplementary file 1 [file jof-08-01315-s001.zip › jof-2056969-supplementary/Supplementary Table S1.pdf]

Supplementary Table 1. List of species used in this study.

Species	Strain	References	Genome URL
Aspergillus niger	NRRL3	[1,2]	https://mycocosm.jgi.doe.gov/Aspni_NRRL3_1/Aspni_NRRL3_1.home.html
Aspergillus nidulans	FGSC A4	[3,4]	https://mycocosm.jgi.doe.gov/Aspnid1/Aspnid1.home.html
Penicillium subrubescens	FBCC1632/CBS132785	[5]	https://mycocosm.jgi.doe.gov/Pensub1/Pensub1.home.html
Trichoderma reesei	QM6a	[6,7]	https://mycocosm.jgi.doe.gov/Trire_Chr/Trire_Chr.home.html
Phanerochaete chrysosporium	PR-78	[8]	https://mycocosm.jgi.doe.gov/Phchr2/Phchr2.home.html
Dichomitus squalens	CBS 464.89	[9]	https://mycocosm.jgi.doe.gov/Dicsqu464_1/Dicsqu464_1.home.html

References

[1] Vesth TC, Nybo JL, Theobald S, Frisvad JC, Larsen TO, Nielsen KF, *et al.* Investigation of inter-and intraspecies variation through genome sequencing of *Aspergillus* section *Nigri.* Nat Genet 2018;50:1688-95. https://doi.org/10.1038/s41588-018-0246-1.

[2] Aguilar-Pontes MV, Brandl J, McDonnell E, Strasser K, Nguyen T, Riley R, *et al.* The gold-standard genome of *Aspergillus niger* NRRL 3 enables a detailed view of the diversity of sugar catabolism in fungi. Stud Mycol 2018;91:61-78.

https://doi.org/10.1016/j.simyco.2018.10.001.

[3] Arnaud MB, Cerqueira GC, Inglis DO, Skrzypek MS, Binkley J, Chibucos MC*, et al.* The *Aspergillus* Genome Database (AspGD): recent developments in comprehensive multispecies curation, comparative genomics and community resources. Nucleic Acids Res 2012;40:D653-D9. https://doi.org/10.1093/nar/gkr875.

[4] Galagan JE, Calvo SE, Cuomo C, Ma L-J, Wortman JR, Batzoglou S*, et al.* Sequencing of *Aspergillus nidulans* and comparative analysis with *A. fumigatus* and *A. oryzae.* Nature 2005;438:1105-15. https://doi.org/10.1038/nature04341.

[5] Peng M, Dilokpimol A, Mäkelä MR, Hildén K, Bervoets S, Riley R*, et al.* The draft genome sequence of the ascomycete fungus *Penicillium subrubescens* reveals a highly enriched content of plant biomass related CAZymes compared to related fungi. J Biotechnol 2017;246:1-3. https://doi.org/10.1016/j.jbiotec.2017.02.012.

[6] Li W-C, Huang C-H, Chen C-L, Chuang Y-C, Tung S-Y, Wang T-F. *Trichoderma reesei* complete genome sequence, repeat-induced point mutation, and partitioning of CAZyme gene clusters. Biotechnol Biofuels 2017;10:1-20. https://doi.org/10.1186/s13068-017-0825-x.

[7] Martinez D, Berka RM, Henrissat B, Saloheimo M, Arvas M, Baker SE*, et al.* Genome sequencing and analysis of the biomass-degrading fungus *Trichoderma reesei* (syn. *Hypocrea jecorina*). Nat Biotechnol 2008;26:553-60. https://doi.org/10.1038/nbt1403.

[8] Ohm RA, Riley R, Salamov A, Min B, Choi I-G, Grigoriev IV. Genomics of wood-degrading fungi. Fungal Genet Biol 2014;72:82-90. https://doi.org/10.1016/j.fgb.2014.05.001.

[9] Casado López S, Peng M, Daly P, Andreopoulos B, Pangilinan J, Lipzen A*, et al.* Draft genome sequences of three monokaryotic isolates of the white-rot basidiomycete fungus *Dichomitus squalens.* Microbiol Resour Announc 2019;8:e00264-19. https://doi.org/10.1128/mra.00264-19.
